# Insights Into Genetic and Molecular Elements for Transgenic Crop Development

**DOI:** 10.3389/fpls.2020.00509

**Published:** 2020-05-15

**Authors:** Marcos Fernando Basso, Fabrício Barbosa Monteiro Arraes, Maíra Grossi-de-Sa, Valdeir Junio Vaz Moreira, Marcio Alves-Ferreira, Maria Fatima Grossi-de-Sa

**Affiliations:** ^1^Plant Biotechnology, Embrapa Genetic Resources and Biotechnology, Brasília, Brazil; ^2^Department of Molecular Biology and Biotechnology, Federal University of Rio Grande do Sul, Porto Alegre, Brazil; ^3^Department of Genetic, Federal University of Rio de Janeiro, Rio de Janeiro, Brazil; ^4^Department of Genomic Sciences and Biotechnology, Catholic University of Brasília, Brasília, Brazil

**Keywords:** new biotechnological tools, plant genetic transformation, tissue culture, minimal expression cassette, T-DNA delivery

## Abstract

Climate change and the exploration of new areas of cultivation have impacted the yields of several economically important crops worldwide. Both conventional plant breeding based on planned crosses between parents with specific traits and genetic engineering to develop new biotechnological tools (NBTs) have allowed the development of elite cultivars with new features of agronomic interest. The use of these NBTs in the search for agricultural solutions has gained prominence in recent years due to their rapid generation of elite cultivars that meet the needs of crop producers, and the efficiency of these NBTs is closely related to the optimization or best use of their elements. Currently, several genetic engineering techniques are used in synthetic biotechnology to successfully improve desirable traits or remove undesirable traits in crops. However, the features, drawbacks, and advantages of each technique are still not well understood, and thus, these methods have not been fully exploited. Here, we provide a brief overview of the plant genetic engineering platforms that have been used for proof of concept and agronomic trait improvement, review the major elements and processes of synthetic biotechnology, and, finally, present the major NBTs used to improve agronomic traits in socioeconomically important crops.

## Background

Climate change, an increasing world population, and genetic erosion are the main factors indicating a need to improve crop adaptation, tolerance, and productivity. There is a continuing requirement for novel cultivars better adapted to different biomes with improved tolerance to biotic and abiotic stresses and superior yield and quality (Arzani and Ashraf, 2017). Conventional plant breeding, despite being a slow and usually difficult process, has made great contributions over the years. This method has been used mainly to add one simple trait to an otherwise ideal variety/cultivar. In contrast, genetic engineering has provided a complementary tool, allowing horizontal introduction of desirable genes for traits of interest to crop plants. The partnership between genetic engineering tools and conventional plant breeding programs has accelerated accurate and efficient crop improvement. To date, the most successful genetically modified (GM) organism (GMO) traits are of only two types, Bt and herbicide resistance, and transgenic tools are either not effective or not necessary for the improvement of some quantitative traits in crop plants. Nonetheless, the development of new biotechnological tools (NBTs) increases agricultural sector competitiveness in internal and external markets (Limera et al., 2017).

Plant breeding using genetic engineering has allowed the development of several elite cultivars with different agronomic traits. Many plant species have already had their genomes manipulated, and several species-specific transformation protocols are available. Genetic transformation mediated by *Agrobacterium tumefaciens*, biolistic methods, and a combination of both techniques are the most common ways to introduce heterologous DNA (Altpeter et al., 2016). The nuclear genome, until recently, was the main target, however, given the possibility of modification of the chloroplast genome and the advantages of this approach, both genomes have now been engineered (Verma and Daniell, 2007; Jin and Daniell, 2015). Current genetic engineering tools allow the introduction of any DNA sequence from any organism, for example, exogenous genes of interest and regulatory elements to drive the expression of endogenous genes. Regardless of the method of transformation, the integration of this exogenous DNA occurs randomly in the genome as single or multiple copies. The randomness of the insertion and the presence of multiple copies can cause undesirable effects, such as insertion within an important operon, which results in off-target effects. Therefore, the transformation and tissue culture methods and any DNA sequence present in the binary vector or transgene that will be used in genetic engineering need to be planned and optimized specifically for the species or genotype of interest.

**FIGURE 1:**
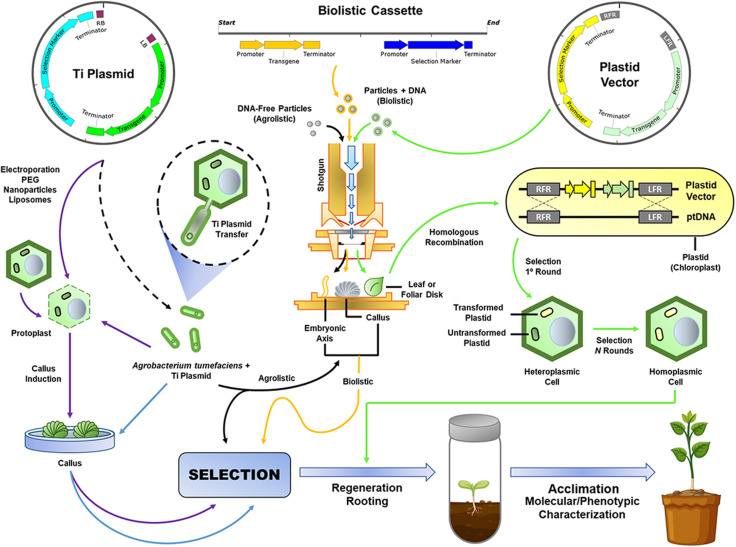
Plant genetic transformation approaches. New biotechnological tools (NBTs) that use mainly the type IV secretion system (T4SS) of *A. tumefaciens*, biolistic, or agrolistic methods for *Ti* plasmid or minimal expression cassette delivery to plant cells (e.g., protoplasts) or tissues (e.g., embryogenic callus or axis, apical meristem, and immature leaf whorl cross-sections), and, finally, transformation of the nuclear or plastid genomes. *Dotted black arrow*: *A. tumefaciens* transformation with the *Ti* plasmid; *purple arrows*: protoplast transformation; *blue arrows*: *Agrobacterium*-mediated callus transformation; *yellow arrows*: biolistic transformation; *black arrows*: agrolistic transformation; *green arrows*: plastid transformation; *ptDNA*: plastid genomic DNA; and *Ti Plasmid*: tumor-inducing plasmid.

In this work, we have provided a brief overview of the plant biotechnological platforms that have been used to develop proofs of concept (hypothesis testing) and improve several agronomic traits (Figure 1). Thus, we reviewed the major elements used for genetic engineering, such as (i) gene constructs (genes of interest, transcriptional promoter sequences, transcriptional terminator sequences (TTS), enhancer and intron sequences, selection marker genes, reporter genes, signal peptides (SPs), and binary and alternative vectors); (ii) plant transformation methods (*Agrobacterium*-mediated T-DNA delivery, biolistic-mediated DNA delivery, agrolistic methods, chloroplast genetic engineering, alternative methods for plant transformation, and clean-gene technology), (iii) and approaches to regulating gene expression [overexpression, gene stacking, RNAi-mediated downregulation of genes, fine-tuning of miRNAs to improve agronomic traits, clustered regularly interspaced short palindromic repeats (CRISPR)/CRISPR-associated protein-9 nuclease (Cas9)-mediated genome editing, CRISPR/dCas9-mediated transcriptional regulation, CRISPR/Cas13a-mediated RNA editing, and CRISPR-ribonucleoprotein (RNP)-based DNA/RNA editing]; (iv) the major existing problems (transgenic versus non-transgenic approaches, plant tissue culture, and genotype-phenotype relationship); and (v) future perspectives on improving desirable agronomic traits in important crops.

## Gene Constructs

### Genes of Interest (GOIs)

New and more robust DNA sequencing platforms have revolutionized plant biotechnological research in recent years (Shendure et al., 2017). Strong and productive research teams, through the sequencing of whole genomes (nuclear, chloroplast, and mitochondrial DNA), exomes, transcriptomes, methylomes, miRNAs and other small RNAs, and translatomes in association with powerful bioinformatic tools and sophisticated molecular biology methods, have obtained a great deal of useful information. In this context, innumerable elements of genetic engineering have allowed the intense exploration of networks by functional genomics in several plant species (both model plants and crops). Gene functions have been revealed and, in some instances, associated with important agronomic traits. Given all this knowledge and expertise, several agronomic traits have become possible targets for improvement, initially using conventional plant breeding assisted by molecular markers and later by genetic engineering based on transgenic approaches. The overexpression and knockdown of genes with functions associated with desirable phenotypes or agronomic traits have made it possible to develop plants with improved characteristics. Currently, several other specific strategies applied to GOIs are also used, such as overexpression of transcription factors (TFs) (e.g., DREB and AREB to improve abiotic stress tolerance), fine-tuning of miRNA, genome editing (using CRISPR/Cas9 or Cpf1), and transcriptional activation or repression (using CRISPR/dCas9 or dCpf1) (Altpeter et al., 2016; Lowder et al., 2017). With respect to the origin of GOIs, both cisgene (gene transference between sexually crossable species or the same species) and transgene (between sexually non-crossable species) methods are used in plant improvement since an orthologous gene in a species of interest may not have the same functional effect as the GOI or show significant expression (Ribeiro et al., 2017). Notably, a putative GOI is often found in multiple copies (paralogs) in the genome; thus, it is recommended to carefully choose between them based on criteria such as expression level, gene structure, and presence of conserved domains. In addition, codon usage optimization for dicots or monocots has been important for improving the translation efficiency of GOIs in specific crops (Murray et al., 1989; Barahimipour et al., 2015). Similarly, the GC content of the GOI can improve its expression level; both codon usage and GC content are also determinants of higher mRNA stability and protein accumulation (Sidorenko et al., 2017; Zhang, 2017). In contrast, a high GC content in the 5′-UTR (untranslated region) can reduce ribosome loading and disrupt translation (Kawaguchi and Bailey-Serres, 2005).

Furthermore, only the protein-coding region (typically called the coding sequence or CDS) of the GOI is inserted into the expression cassette for plant transformation. Since the complete genomic sequence of the protein-coding region generally contains several introns as well as the exon sequence, cloning the full-length region is difficult due to the large size of the region. In addition, the posttranscriptional processing of the full-length transcript may be different in the recipient organism, resulting in an undesirable mRNA and protein, and longer GOI sequences are more likely than shorter sequences to be targeted by DNA silencing machinery, reducing transgene stability. However, in some cases, GOIs are used in their original form (including introns, exons, 5′- and 3′-UTRs) due to the presence of endogenous *cis*-regulatory elements or enhancer sequences essential for their expression, translation or stability (Gao et al., 2015; Zhang et al., 2018).

### Transcriptional Promoter Sequences

Promoters are DNA sequences usually 300–1500 nt in length that are located upstream of the 5′-UTR of the gene, and they contain several regulatory elements involved in the spatiotemporal regulation of transcription initiation (Yamamoto et al., 2007). For the successful transcription of any gene, the transcription initiation complex needs to bind to other proteins, such as activators or repressors, to trigger DNA transcription by the RNA polymerase. Activators and repressors are proteins crucial for regulating gene expression. TFs belong to this important class of proteins and have a DNA-binding domain that will recognize short stretches of DNA called *cis*-regulatory elements (Hernandez-Garcia and Finer, 2014). These *cis*-regulatory elements are conserved functional domains essential for binding specific TFs and other regulatory factors needed to initiate, stabilize, and maintain transcription (Hernandez-Garcia and Finer, 2014). The numbers and types of these *cis*-regulatory elements determine transcription efficiency at the level of transcript amount per cell, tissue- or stage-specific expression and transcriptional activation by different stimuli (biotic or abiotic), allowing rapid and robust responses that permit the plant to adapt to new conditions.

The expression of all mRNAs in plants is constantly coordinated with any ecosystem changes or molecular signals. TFs act as major players in transcriptional regulation by interacting with promoter sequences. For example, DRE is a dehydration-responsive element that recruits TFs (e.g., DREB1 and DREB2) that activate the transcription of genes involved in the cold and dehydration responses (e.g., the *RD29A* gene), while ABRE is an ABA-responsive element that recruits other TFs (e.g., bZIP and AREB) that activate the transcription of genes involved in the dehydration and salinity responses (e.g., *RD29B* gene) (Yamaguchi-Shinozaki and Shinozaki, 2006). Similarly, MYC/MYB elements recruit TFs (e.g., MYC2 and the MYB family) that activate the transcription of genes involved in biotic and abiotic stress tolerance (e.g., the *RD22* gene) (Ambawat et al., 2013). The GC box, CCAAT box, and TATA box domains are usually located approximately 10–110 nt upstream of the transcription start site (TSS), and the binding of specific TFs triggers the formation of transcription complexes. Thus, the TSS is responsible for determining the precise site in the promoter where transcription should begin, yielding the primary transcript (primary mRNA), and the beginning of the 5′-UTR region of these primary transcripts.

The successful acquisition of agronomic traits through GOI overexpression is directly related to the expression level of the GOI at a given stage, as a response to a stimulus or in a specific plant tissue. Thus, the choice of promoter contributes to the efficiency of NBT and the accessibility of powerful traits. Currently, synthetic, viral or plant promoters with constitutive, stress-induced (biotic and abiotic), tissue-specific and developmental stage-specific features are available to drive GOI overexpression in several monocot and dicot crops (Basso et al., 2020). GOI overexpression driven by stress-induced, tissue- or stage-specific promoters reduces the probability of yield penalties in crops and negative effects on non-target organisms. For example, strong and constitutive overexpression of the TFs, AREB, and DREB, results in growth delay or a significant yield penalty in several crops (e.g., cotton, sugarcane, wheat, and barley) (Morran et al., 2011). However, drought-inducible promoters, including those of WRKYs, NAC6, LEA3, RD21, RD27, and RD29, have been successfully used for this purpose and are commonly used to drive GOIs associated with salt or drought tolerance (Agarwal et al., 2017). Similarly, plant-pathogen inducible promoters (e.g., *CYP76M7*, *pGRMZM2G174449*, *PR1b*, and *GST* gene promoters) are of great importance for enhanced disease resistance, the effective management of plant diseases and the prevention of yield penalties (Vijayan et al., 2015; Goto et al., 2016; Yang et al., 2017).

In another context, senescence-induced promoters (e.g., SAG29) may be of interest to direct GOI expression in the late stages of plant development, for example, to direct deconstruction of the cell walls of grasses to increase their enzymatic digestibility and, thus, their yield of lignocellulosic ethanol. Similarly, endosperm-specific promoters may be of interest for GOI expression in grains to improve nutritional quality, grain size and shape, or stress tolerance or to produce proteins of interest for storage (Li et al., 2008). Liang et al. (2019) improved folate accumulation in maize and wheat seeds by overexpression using the endosperm-specific promoter to drive the genes responsible for synthesizing the folate precursors pterin and p-aminobenzoate.

However, when a high level of GOI expression is needed to achieve a desirable phenotype (e.g., entomotoxic proteins), the use or discovery of new species-specific promoters that confer high transcript accumulation is indispensable (Ribeiro et al., 2017). Synthetic, viral, and plant promoters have been evaluated, but there are few available sequences, and most of them have been validated in only one plant species and may not work well in other species. In addition, a significant increase in GOI transcription has been obtained by genome editing tools using deactivated nucleases fused to activation domains and guided to promoters. Genome editing technologies can also be used to edit or insert specific *cis*-regulatory elements into promoter sequences to modulate GOI expression levels (Wolter and Puchta, 2018). Further information on using genome editing technology to act on promoter sequences is detailed below.

### Transcriptional Terminator Sequences (TTS)

TTS are conserved sequences composed of *cis*-regulatory elements downstream of the protein-coding region (mRNA 5′- or 3′-UTR), which are recognized by the transcriptional machinery as transcription stop signals and consequently induce decoupling of this machinery from the DNA (Loke et al., 2005). Efficient TTS are associated with improved transcription levels, mRNA polyadenylation (poly-A), and RNA transcript termination. Poly-A signals in the 3′-UTR of plant genes are composed of three major components: *far* upstream elements (*FUE*, uracil-rich sequences) located approximately 100 nt upstream of the poly-A site; *near* upstream elements (*NUE*, adenine-rich sequences) located approximately 25 nt upstream of the poly-A site; and *CSs* (cleavage sites), which are YA dinucleotides (CA or UA) situated within a uracil-rich region located downstream of the *FUE* and *NUE* (Tian and Graber, 2012). mRNA polyadenylation is crucial to mRNA posttranscriptional processing (splicing), stability, nuclear-to-cytoplasmic export, and translation. The most successfully used TTS in plants are T-nos (254 nt in length, from the *nopaline synthase* gene of *A. tumefaciens*), T-35S (226 nt in length, from the *Cauliflower mosaic virus* 35S terminator), rbcS1 or rbcS-E9 (291 nt in length, from the *ribulose-1*,*5-bisphosphate carboxylase* gene, small subunit, of *Pisum sativum*), and T-ocs (196 nt in length; from the *octopine synthase* gene of *A. tumefaciens*). However, plant-specific and gene-original terminators can also be used in some instances, for example, for transcription of the *acetohydroxy acid synthase* (*ahas*) gene as a selectable marker gene (Aguiar et al., 2012). In addition, it was also observed that two terminator sequences in tandem (e.g., T-nos + T-35S) could enhance the transcription level (Diamos and Mason, 2018; Yamamoto et al., 2018). Beyene et al. (2011) suggested that a double TTS improved the stability of transgene expression by causing more efficient transcription termination and reducing posttranscriptional gene silencing (PTGS) of the target gene. Similarly, Diamos and Mason (2018) showed that combining two or three terminators in tandem increased the expression level up to 25-fold.

### Intron-Mediated Enhancement and Enhancer Sequences

Introns are non-coding sequences present in primary transcripts that are removed by splicing before the translation of the coding sequence (exons). However, some intron sequences have additional functions useful in genetic engineering, such as acting in intron-mediated transcription enhancement and improving translation efficiency (Laxa, 2017). Additionally, some introns can be associated with strong, constitutive, tissue- and developmental stage-specific expression of endogenous genes or transgenes (Liao et al., 2013). These introns contain specific motifs (e.g., TTNGATYTG) and must be used in the correct orientation, inside the 5′-UTR, and next to the TSS (Gallegos and Rose, 2015; Rose et al., 2016). Gallegos and Rose (2017) showed an unexpected role of these introns in determining a new TSS. In monocots, splicing is required to remove this intron from the primary transcript, while, in dicots, splicing is not essential (Clancy and Hannah, 2002).

The *Adh1*, *Sh1*, *Bz1*, *Hsp82*, *Act1*, and *GapA1* introns from maize or rice genes are those most commonly used to improve transcription levels in monocots, while the *rbcS*, *ST-LS1*, *Ubq3*, *Ubq10*, *PAT1*, and *atpk1* introns from petunia, potato, or *A. thaliana* genes are the most common in dicots (Gallegos and Rose, 2015; Laxa, 2017). For example, the *Ubi1* intron (520 nt in length and isolated from the *Ubiquitin 1* gene of maize) is widely used to enhance transcription from the *Ubi1* promoter in transgenic monocots (e.g., sugarcane, rice, sorghum, and *Setaria viridis*). In addition, the *Ubq10* promoter (634–1104) has been enhanced by the *Ubq10* intron (64 nt in length, from the *polyubiquitin 10* gene of *A. thaliana*) in transgenic dicots (e.g., soybean and *A. thaliana*). Additional strategies based on the insertion of introns into the protein-coding regions of selectable marker (e.g., *bar/pat* and *hptII*) and reporter genes [e.g., *green fluorescent protein* (GFP)] serve to avoid translation in prokaryotes; the *ST-LS1* (from the *ST-LS1* gene of potato) and *ADH1* (from *alcohol dehydrogenase-1* gene of maize) introns are the most commonly used for this purpose in both monocots and dicots.

Unlike introns, enhancers are non-coding DNA sequences commonly found within the promoter sequence upstream of the TSS or in the 5′- or 3′-UTR; they can bind multiple TFs to activate the expression of genes located up- or downstream. In addition, enhancers display conserved TF binding motifs; regulate enhancer RNA expression, chromatin accessibility, and histone modifications; have reduced DNA methylation levels; and physically interact with their target genes (Weber et al., 2016). For example, a maize *Hepta-repeat* enhancer located 100 kb upstream of the *booster1* gene improves its expression (Belele et al., 2013), while a 52 nt enhancer in the 3′-UTR of the *SELF PRUNING 5G* gene is essential for enhanced expression of this gene in tomato (Zhang et al., 2018). Mitsuhara et al. (1996) showed that the first intron of a gene for phaseolin from *Phaseolus vulgaris* and the 5′-UTR sequence (G-free sequence; Ω sequence) of *Tobacco mosaic virus* enhanced 35S promoter activity. Benfey et al. (1990) showed that the upstream region from -343 to -46 of the 35S promoter also acts as an enhancer sequence, and this result was subsequently confirmed by high expression levels when genes were driven by the 35S-enhanced promoter (Beringer et al., 2017). Davies et al. (2014) identified and showed that the *Sugarcane bacilliform virus* enhancer improved transcript levels in transgenic maize and that multiple tandem copies were more effective than a single copy in increasing transcription. Similarly, Maiti et al. (1997) showed that the FLt promoter (from *Figwort mosaic virus*) with a double enhancer domain improved the transcript level twofold compared to FLt with a single enhancer domain. Overall, intron and enhancer sequences have great potential for application in genetic engineering, but the low number of validation studies supporting the use of these sequences in combination with typical promoters or in specific crops has contributed to their restricted and uncertain use.

### Selectable Markers

The challenge of genetic transformation is to insert the DNA of interest into the nuclear genome of the cell and then to select this transformed cell and potentiate its regeneration. This selection occurs through the addition of selective agents to the *in vitro* culture medium (e.g., hygromycin, kanamycin, geneticin, glyphosate, glufosinate-ammonium, and imazapyr) followed by several subculture steps and the use of hormones. Selection starts after a coculture period in darkness or low light, which can be increased gradually to potentiate selection (reducing the escape number) and decrease tissue oxidation (Basso et al., 2017). Most plant species or genotypes have predefined recommendations for the best selective agent to improve their selection and regeneration and to increase transformation efficiency. For example, for sugarcane transformation, glufosinate-ammonium and geneticin are recommended (Dong et al., 2014; Basso et al., 2017); for cotton and soybean, imazapyr (Li et al., 2017; Ribeiro et al., 2017); and for *Setaria viridis* and *S. italica*, hygromycin (Van Eck, 2018; Santos et al., 2020). Glufosinate-ammonium is excellent for *A. thaliana*, mainly due to the practicality of selection *in vivo* (through spraying of plants with an already well-defined concentration of this agent). Hygromycin is also a good selective agent for *A. thaliana*, but seed screening must be performed *in vitro*, which makes selection more laborious (Harrison et al., 2006). Thus, selecting the best marker gene for use in the plant species of interest is one of the first steps before beginning the design and synthesis of the transformation vector. Selection efficiency can be improved using strong species-specific promoters (e.g., the CaMV 35S promoter in dicots, rice or maize ubiquitin, or actin promoters in monocots), the insertion of an optimized Kozak sequence before the start ATG, and optimized codon usage in the selection marker gene.

The selection of transgenic plants is based on a gene product (mRNA and protein/enzyme) that usually confers resistance to selective agents (e.g., *bar* or *pat* genes confer resistance to glufosinate-ammonium, the *nptII* gene confers resistance to geneticin or kanamycin, the *hptII* gene confers resistance to hygromycin, *cpt-cp4 epsps* confers resistance to glyphosate, and the engineered *ahas* or *als* gene confers resistance to the imidazolinone, sulfonylurea, and triazolopyrimidine herbicide classes). Positive selection occurs when selectable markers confer a selective advantage on transformed cells without causing injury or death of non-transformed cells, while negative selection occurs via growth inhibition and death of non-transformed cells.

The *uidA/gus* (β*-glucuronidase*), *manA* (*phosphomannose isomerase*), *xylA* (*xylose isomerase*), *PTXD* (*phosphite oxidoreductase*), and *DOG^*R*^1* (*2-deoxyglucose-6-phosphate phosphatase*) genes isolated from microorganisms are among the main markers for positive selection in plant tissue culture (Izawati et al., 2015; Nahampun et al., 2016). In contrast, the *nptII*, *hptII*, and *CmR* genes are some examples of negative selection markers that confer resistance to antibiotics (geneticin/kanamycin, hygromycin, and chloramphenicol, respectively) that block ribosome activity and inhibit protein synthesis. The principal concerns of using these selection markers are the occurrence of horizontal gene transfer to non-target organisms and the potential toxicity to organisms that consume these transgenic plants.

Selection markers that confer high tolerance to certain herbicides, such as the *bar* (or *pat*), *ahas* (or *als*), and *cpt-cp4 epsps* (or *aroA*) genes, have been widely used to select plants with transgenic nuclear genomes, mainly due to their relatively low rate of escape. In chloroplast genome transformation, the *aadA* gene (streptomycin 3′-adenylyltransferase), which confers resistance to spectinomycin and streptomycin, is widely used for transgenic chloroplast selection. However, several strategies have been developed to recover marker-free transgenic plants, although most have limitations and low efficiency. The cotransformation strategy, which uses two different binary vectors (carrying the GOI and selection marker separately) followed by segregation steps for the elimination of the transgene containing the selection marker, has been used but showed very low efficiency (Yau and Stewart, 2013). In addition, cotransformation with one binary vector containing two T-DNAs or one T-DNA with two right/left borders has been used to independently transfer and integrate the GOI and selection markers into the genome. Site-specific recombination, transposon techniques, positive-negative selection with a cotransformation system, and genome editing are examples of the techniques used to remove selection markers from transgenic plants (Yau and Stewart, 2013).

### Exogenous and Endogenous Reporter Genes

Reporter proteins are used in genetic engineering to facilitate molecular biology studies and minimize the manipulation of plants. For this strategy, features such as ease of use, low cell toxicity, robustness, and high signal are important for success. The exogenous reporters most used are GFP (or eGFP), *beta-glucuronidase* (*uidA*/GUS), *luciferase* (LUC), *yellow fluorescent protein* (YFP), and *red fluorescent protein* (RFP, mCherry or DsRed2), while *phytoene desaturase* (PDS) is the endogenous reporter gene most used in plants to evaluate RNAi assays. Their applications are diverse and include initial screening of regenerating cells or plants to distinguish transgenic from non-transgenic, reporters in agroinfiltration and transient expression assays, screening of RNA silencing suppressors, determining subcellular localization (e.g., fusion of the protein of interest to a reporter protein and detection using confocal or fluorescence microscopy), examining gene expression levels (e.g., evaluation of a promoter sequence or fusion to a protein of interest), and intracellular protein trafficking. In addition, the tagging of endogenous genes with reporter proteins (e.g., GFP and LUC) using new genome editing tools is being explored to support functional genomics studies (Fetter et al., 2015). The *PDS* gene has been widely used as a proof of concept for topical dsRNA delivery systems in plants, including nanostructures and stabilizing agents of RNA and dsRNA complexed in nanoparticles; RNAi-based gene silencing in transgenic plants; virus-induced gene silencing (VIGS); and genome editing (Chen et al., 2018; Naim et al., 2018). Disruption of the *PDS* gene typically results in albinism and dwarf phenotypes by impairing chlorophyll, carotenoid, and gibberellin biosynthesis (Qin et al., 2007).

Enhanced GFP (eGFP) from the jellyfish *Aequorea victoria* is a mutated version of GFP; it differs in a few amino acids that result in higher fluorescence, and its excitation wavelength is 489 nm and emission 509 nm. Currently, eGFP is widely used, mainly as a reporter to find and validate new promoter sequences, to screen transformed cells or to fuse with proteins of interest. Other reporter proteins originating from GFP mutants with different fluorescence spectra are also known, such as *yellow*, *blue* (BFP), and *cyan fluorescent proteins* (CFP). For example, YFP is used in bimolecular fluorescence complementation (BiFC) assays to study molecular interactions, while CFP is used to monitor physiological processes, visualize protein localization, and detect transgene expression.

Similarly, the *uidA* gene encodes the *hydrolyase*β*-glucuronidase* (GUS) enzyme, which has as one of its histochemical substrates X-Gluc (5-bromo-4-chloro-3-indolyl-beta-D-glucuronic acid, cyclohexylammonium salt). Constitutive or transient expression of GUS results in X-Gluc degradation, producing colorless glucuronic acid and a visible, intensely blue precipitate. This gene is also widely employed in genetic engineering due to its ease of use (e.g., exposing the tissue of the transgenic plant to the X-Gluc substrate and incubating the samples overnight at 37°C), rapid (∼24 h) detection, relatively high accuracy and ease of observation and interpretation. However, GUS activity is usually measured or visualized in tissues excised from the transgenic plants and exposed to a buffer containing the X-Gluc substrate.

The *lacZ* gene, which encodes the β-galactosidase enzyme and is widely used as a reporter in cloning vectors (e.g., pGEM^®^ -T easy vector; Promega) with the substrate X-Gal (5-bromo-4-chloro-3-indolyl-β-D-galactopyranoside), has also been tested in plants. If genes are successfully cloned into the multiple cloning sites (MCS) of these vectors, the *lacZ* gene is truncated, and no β-galactosidase transcript is produced. After cloning, the vector is transfected into competent *Escherichia coli*, and the transformed cells are plated on selective medium containing antibiotics and X-Gal. Blue bacterial colonies contain DNA without the fragment of interest (empty vector), while white colonies indicate successful cloning.

### Signal Peptides (SPs) to Target Proteins to Specific Organelles

After DNA transcription, mRNA is processed by splicing, transported to the cytoplasm, and translated by ribosomes, which are either free or associated with the endoplasmic reticulum (ER). Two basic targets are known: the posttranslational (targeting the nucleus, mitochondria, chloroplast, ER, and peroxisomes) and cotranslational or secretory (targeting the ER, Golgi apparatus, lysosomes, plasma membrane, and secreted vesicles) pathways (Lee et al., 2014). After proteins are synthesized in the cytoplasm, they can be targeted by SPs, nuclear localization signals (NLS; e.g., from SV40 large T-antigen and nucleoplasmin protein) or transmembrane domains to the places where they will act. In contrast, proteins without an SP are permanently retained in the cytoplasm. SPs are short (∼7–36 amino acids), hydrophobic, and positively charged amino acid residues containing at their C-terminal ends a signal peptidase; they are present mostly at the N-termini of proteins and sometimes at their C-termini. Additionally, depending on which organelle is targeted (e.g., chloroplast, vacuole, or mitochondria; not the ER), a protein may have two or more SPs (Christensen et al., 2005). These SPs are recognized by the import machinery that selectively transports proteins to organelles and mediates their intracellular translocation through the membrane. Then, in most instances, the SP is cleaved off the protein by organelle-specific peptidases after it has reached its final destination.

SPs are currently in great biotechnological demand to target heterologous enzymes or proteins of interest to specific plant organelles to increase the amount of protein per cell by reducing its cytotoxic effects. In addition, the use of SPs with heterologous enzymes can contribute to efficient targeting and consequently enhance their activities in the desired organelle. For example, the plant EPSPS enzyme acts through the shikimate pathway in the biosynthesis of aromatic amino acids, specifically in the chloroplast. In contrast, glyphosate-resistant heterologous EPSPS (from *Agrobacterium* spp. strain CP4) efficiently targets soybean chloroplasts in combination with the N-terminal petunia chloroplast transit peptide (CTP). The *ctp-cp4 epsps* fusion improves the targeting of the enzyme to the chloroplast and the resistance of the transgenic plants to glyphosate (Hammer et al., 2013). Similarly, the high efficiency of the nucleases used in plant genome editing depends on their nuclear localization, and for this, their heterologous expression is performed with NLS fusions at the N-terminal and C-terminal ends (Liang et al., 2017). In contrast, the KDEL (H6KDEL) SP is located on the C-terminal end of the amino acid structure of a protein and prevents protein secretion from the ER. In this way, it is widely used to target heterologous proteins or antibodies to the ER of biofactory plants (e.g., lettuce, tobacco, and *Nicotiana benthamiana*). Similarly, the use of N-terminal γ-zein proline-rich sequences, (VHLPPP)_8_, to target heterologous proteins to the ER and protein bodies increases protein accumulation in seeds (Torrent et al., 2009). Two other examples, ConA and Endochitinase A C-terminal SPs from *Canavalia ensiformis* and *Nicotiana tabacum*, target proteins to the vacuole, while ARA12 from the subtilisin-like serine proteases of *A. thaliana* targets proteins to the apoplast. In summary, there are numerous known SPs with some specific features that have already been validated for targeting heterologous proteins to different organelles in several plant species. Ng et al. (2016) confirmed that the fusion of SPs (e.g., citrine-NLS and citrine-peroxisomal targeting signal) with proteins of interest facilitates their targeting after intracellular delivery, and Shen et al. (2017) developed the RC2 optimized transit peptide for effective targeting of diverse foreign proteins into rice chloroplasts. Intriguingly, Hou et al. (2019) showed that fusion of Cry proteins with *E. coli* maltose-binding protein (MBP) enhanced their activity against western corn rootworm, probably due to increased solubility of the MBP-Cry8Hb fusion in the rootworm midgut.

### Binary and Alternative Vectors

Since the elucidation of the type IV secretion system (T4SS) involved in *Agrobacterium*-mediated plant transformation, several improved genetic transformation protocols based on *Agrobacterium* or agrolistic methods have been established for several plant species. Because *Ti* plasmids are very large (∼200 kb in length), they are difficult to handle *in vitro*, complicating the removal or insertion of any DNA into specific sites. In addition, their backbone was engineered to allow replication in both *E. coli* and *Agrobacterium* sp. using an origin of replication that allows either a high (e.g., ColE1 for *E. coli*) or low (e.g., pVS1 for *A. tumefaciens*) copy number per bacterial cell. In contrast, the *vir* genes required for T-DNA transference are allocated into cointegrated *Ti* plasmids or anchored in the *Agrobacterium* genome (Gelvin, 2010). Another strategy, which is currently underused, is based on small “intermediate” or “shuttle” plasmids engineered to introduce any desirable DNA sequence into the T-DNA of the endogenous *Ti* plasmid. In addition, the binary system has been enhanced to improve the genetic transformation efficiency of different plant species. The development of superbinary vectors harboring additional *vir* genes and ternary vectors containing an additional helper plasmid with an increased number of *vir* gene copies has shown promising results (Anand et al., 2018; Che and Anand, 2018). Reduced T-DNA length and component optimization are crucial requirements to prevent its breakage during transfer and to increase the transformation efficiency and stability of the transgene. Indeed, the design, synthesis, and assembly of a binary or alternative transformation vector are not rapid, simple, or inexpensive activities. Thus, vectors with MCS or restriction enzyme sites flanking the major transcription units are currently being engineered. This strategy allows the sequences of interest (e.g., exchanges of the promoter, terminator, GOI, or selection marker gene) to be changed and the vectors to be reused. Another currently adopted alternative is the use of optimized, open-source, and free-to-operate binary vectors. For example, the pCAMBIA vectors are improved binary vectors used for plant transformation. This system has several advantages, such as small size (7–12 kb in length), suitability for Gateway^®^ technology, high-copy-number origin of replication in *E. coli*, convenient restriction sites for sequence exchange, multiple selectable markers for both bacteria and plants, methods for constructing reporter gene fusions (e.g., *gus* and *gfp*), and an adequate MCS for inserting genes of interest and popular promoters. However, these traditional vectors also have some important drawbacks; for example, their components may not be optimized for specific cases (e.g., codon usage, GC content, or optimal Kozak sequence for a specific plant species) or their components may not be ideal for a specific purpose (e.g., promoter, terminator, selection marker, or reporter gene). To overcome these limitations, researchers have preferred to synthesize new, simple, and optimized vectors for each specific case. Currently, some companies provide synthesis and cloning services, providing optimized vectors within 2–4 months, but their prices remain high.

The identification of genome regions with high expression levels can provide DNA sequences for the engineering of minimal expression cassette (MC) borders. The insertion of engineered transgenes into the plant genome may occur with greater specificity in these hot spots, thereby increasing the transcription level and augmenting the stability of GOIs by minimizing the position effect. In recent years, the generation of several independent events and the selection of those with the highest level of expression have been preferred over identifying hot spots and directly inserting by site-specific recombination. Additionally, binary vectors carrying engineered T-DNA for insertion based on transposition have been developed for plant transformation. These long T-DNA fragments typically contain (i) an *Ac-transposase* (*Tpa*) gene responsible for the initiation of transposition, (ii) a MC carrying specific elements flanked by transposable dissociation motifs (*Ac*), and (iii) a selection marker gene. The T-DNA is delivered by *A. tumefaciens* or biolistics, and the Tpa enzyme is translated (transiently or after integration into the plant genome), binds to the *Ac* motifs and drives the site-specific integration of the MC into the plant genome. Then, the *Tpa* and selectable marker genes are removed from the plant genome using Mendelian segregation, while the MC is stably maintained after transposition (Jin et al., 2003). Thus, this strategy may not be compatible with vegetatively propagated plants. Although using transposition vectors results in a lower transformation efficiency, a low frequency of single-copy integration, and a low number of backbone-free transformation events compared to conventional T-DNA, a higher expression level of transgenes inserted by transposition has been observed (Yan and Rommens, 2007).

Nuclear matrix attachment regions (MARs) are A/T-rich DNA sequences of approximately 400 bp found at the borders of non-transcribed genes that mediate the structural organization of chromatin within the nucleus (Dolgova and Dolgov, 2019). MAR sequences have been used to flank MCs to reduce transgene silencing, improve expression stability (Zhang et al., 2004; Li et al., 2008), and enhance transformation efficiency (Petersen et al., 2002). Diamos and Mason (2018) showed that combining dual terminators in tandem with MARs increased expression up to 60-fold compared with the terminator alone. However, MAR remains unusual in plant engineering because these sequences act in plant tissue- and species-specific manners (Ascenzi et al., 2003); in some cases, they may result in lower expression levels (Breyne et al., 1992) or may not provide satisfactory stability (Schöffl et al., 1993).

## Transformation Methods

### Agrobacterium-Mediated T-DNA Delivery

*Agrobacterium* spp. (*Rhizobiaceae* family) are gram-negative bacteria capable of inducing crown gall (*A. tumefaciens* and *A. vitis*), hairy root disease (*A. rhizogenes*), and cane gall (*A. rubi*) in several plant species. A compatible interaction between *A. tumefaciens* and its host plant results in T-DNA delivery mediated by the T4SS into plant cells. Low-molecular-weight phenolic compounds, amino acids, and sugars present at the site of infection serve as primary signaling molecules for the recognition and activation of virulence (*vir*) genes. In addition to the *vir* genes located in the bacterial genome, which are involved in the initial infection process, six groups of genes are considered essential for the T4SS (*vir*A, *vir*B, *vir*C, and *vir*D) or increase its efficiency (*vir*E and *vir*G). Thus, the T-DNA originating from a tumor (*Ti*)- or root (*Ri*)-inducing plasmid is translocated from the plant cytoplasm to the nucleus and randomly integrated by recombination into the genomic DNA. The T-DNA sequence is typically delimited by two direct 25 bp repeats (the left and right T-DNA borders), which are essential for the recognition of T-DNA by the *vir*D and *vir*E proteins and for its consequent release from the *Ti* plasmid. Then, the T-DNA is transferred into the plant nucleus by single-stranded DNA (ssDNA)-associated virulence proteins encoded by *Agrobacterium* (Gelvin, 2010). Finally, the expression and translation of the oncogenes present in the T-DNA in the transfected plant cells directly interfere with gene expression and phytohormone biosynthesis.

Before this method was developed, the engineering of the *Ti* plasmid allowed the successful use of *Agrobacterium* sp. as a system of biotechnological interest, allowing DNA sequence delivery to totipotent cells in several plant species. After coinoculation and several stages of selection and regeneration of transgenic cells, it is possible to achieve a non-chimeric transgenic plant with desirable agronomic characteristics. Additionally, the advancement of generations increases the integration of the T-DNA, and the expression of the transgene becomes increasingly stable (Ribeiro et al., 2017; Ribeiro et al., 2020).

For this process, it is necessary to engineer the T-DNA into a binary vector, replacing the tumor-causing genes (resulting in a “disarmed” *Ti* plasmid) with promoters, genes of interest, and transcription terminator sequences. The main advantage of this method is its high rate of single transgene insertion. Furthermore, the efficiency of transformation can be enhanced by the use of bacterial strains with different degrees of virulence (e.g., GV3101, C58C1, EHA105, LBA4404, and AGL1 are some of the *A. tumefaciens* strains most commonly used in plant transformation), higher tolerance to recalcitrant tissues or better adaptation to the desired plant species. EHA105, AGL1, and LBA4404 are considered hypervirulent strains, most likely due to increased induction of the *vir* genes. These strains are recommended for the transformation of recalcitrant or monocot plants, while the milder strains are most often recommended for non-recalcitrant dicotyledonous plants. Notably, using a hypervirulent strain might reduce transformation efficiency in some plants (e.g., tomato cv. Micro-Tom) compared to that using other *Agrobacterium* strains, such as GV3101. In addition, the T4SS can be activated or enhanced by the direct addition of acetosyringone (a phenolic of natural or synthetic origin) to the *Agrobacterium* growth medium (e.g., YEB or LB) and liquid or solid coinoculation medium. Another preconditioning step can be performed by gently incubating (in the dark at 22°C for 12–16 h) the *Agrobacterium* cells in *Agrobacterium* (AB) minimal medium supplemented with acetosyringone (Armstrong et al., 2015; Basso et al., 2017).

### Biolistic-Mediated DNA Delivery

Biolistic-mediated transformation emerged in 1987 as an alternative to protoplast transformation. Unlike *Agrobacterium*-mediated T-DNA delivery, the biolistic transformation method (particle bombardment or gene gun) allows the direct introduction of any DNA sequence into the plant genome. For this, the target DNA sequence (binary vector or MC) is dehydrated and complexed with small (0.6–1 μM in diameter) gold or tungsten particles (microcarriers). Then, the microcarriers are deposited on the membranes, accelerated with helium gas to a high velocity using a PDS-1000/He^TM^ or similar system, and bombarded against totipotent plant tissue. Inside the cells, if the DNA has not reached the nucleus, it is disassembled and directed to the nucleus, where it will integrate randomly into the nuclear genome. Gold particles are recommended due to their greater uniformity of size and lack of toxicity (inertness) to plant cells. However, backbone insertion into the plant genome is undesirable, and for this reason, the use of MCs is recommended (Taparia et al., 2012). The main advantage of the biolistic method is the possibility of direct transformation of tissues such as pollen, embryos, meristems, and morphogenic cell cultures regardless of plant species. In addition, larger or multiple transgenes can be used with this transformation method. However, the use of very long sequences increases the risk of DNA breakage during delivery. The insertion of multiple copies into the genome is also undesirable because it is prone to instability over successive generations and increases the possibility of DNA integration in important intragenic regions. It also has a high financial cost, as the regulation of these transgenic plants for commercial use is expensive. Using an optimized concentration of DNA in each shot is important to reduce the insertion of multiple copies. For example, Kim et al. (2012) efficiently produced transgenic sugarcane with a low copy number from embryogenic callus bombarded with 12–50 ng of MCs per shot. On the other hand, two or three shots in the same tissue may be used to increase transformation efficiency. For some recalcitrant plant species (e.g., cotton and sugarcane), the biolistic method has been used with reasonable transformation efficiency (Ribeiro et al., 2017), while for some plant species, transformation mediated by *A. tumefaciens* has been more satisfactory. The damage caused to the bombarded tissue (e.g., cell disruption) can be minimized by incubating the tissue in an osmotic medium for a few hours before the procedure.

### Agrolistic-Mediated Plant Transformation

The agrolistic method uses the advantages of *A. tumefaciens* in combination with the high efficiency of DNA delivery achieved with biolistics, allowing increased transformation efficiency. However, it has been used most often for the transformation of recalcitrant plants, such as cotton and soybean. In addition, biolistics using microcarrier particles without DNA can be used to cause minor and superficial injuries. Then, the injured tissue can be cocultivated with the desired *A. tumefaciens* strain. For example, microprojectile bombardment before cocultivation with *A. tumefaciens* enhanced the transformation efficiency of tobacco leaves and sunflower apical meristems by at least 100-fold when compared with that of biolistics alone. Similarly, Abdollahi et al. (2009) overcame the physical barrier of the thick rapeseed microspore wall through microspore bombardment with microprojectile particles without DNA and coinoculation with *A. tumefaciens* culture. However, because biolistic methods are onerous, alternatives have been adopted to serve the same purpose, such as thermal shock before coinoculation, vacuum infiltration, cocultivation in Petri dishes containing coculture medium or hydrated filter paper, needle injury, or tissue sonication (Dong et al., 2014).

### Chloroplast Genetic Engineering

Transformation of the chloroplast genome offers important advantages over that of the nuclear genome in the development of biotechnological tools (Adem et al., 2017). This strategy has been exploited to produce biopharmaceuticals (e.g., vaccines, human serum albumin, peptides, proteins, and antigens), control insect pests, and engineer herbicide, drought, and pathogen resistance in model plants or economically important crops. Bally et al. (2016) showed that the overexpression of dsRNA targeting the acetylcholinesterase gene was more stable and effective in the control of *Helicoverpa armigera* when integrated into the chloroplast genome of *N. benthamiana* than when integrated into the nuclear genome.

Notably, chloroplasts do not contain RNA interference machinery and epigenetic mechanisms that could interfere with the expression of heterologous transgenes (Zhang, 2015). Transgene insertion into the genome typically occurs via homologous recombination, so the position effect of the transgene is minimized, and protein accumulation is stable. The high number of chloroplasts in each cell and the polyploidy of its genome allow the insertion of several transgene copies in a single cell, resulting in uniform and strong protein accumulation levels (Grevich and Daniell, 2005). Recombinant protein accumulation in transgenic chloroplasts can lead to less cytotoxicity in plant cells than cytosolic protein storage. In addition, the maternal inheritance of chloroplasts in most crops prevents the transgene from being transmitted via pollen to other plant species (e.g., weeds or sexually compatible crops) and additionally reduces any eventual entomotoxic effects on pollinating or non-target insects. Furthermore, transgenic plants may optionally be generated without any antibiotic resistance marker gene, and because chloroplasts support the formation of disulfide bonds, they represent excellent biofactories for mammalian proteins that require this form of folding (Bally et al., 2008; Mohammadinejad et al., 2019).

Typical vectors for chloroplast genome transformation contain the GOI, a selectable marker gene (e.g., *badh*, *aadA*, *neo*, and *aphA6*) driven by an organelle-specific promoter (e.g., rrn, psbA, rbcL, and 16S rRNA) and 5′- and 3′-UTRs (e.g., from the *psbA* or *rbcL* transcripts) that enhance transcription and translation. This expression cassette must be flanked on the left and right by two genomic regions (e.g., intergenic regions between the *trnA* and *trnI* genes) to allow site-specific insertion by homologous recombination (Verma and Daniell, 2007). Vector delivery is performed by the biolistic method in leaf segments, which are then placed on selection medium and periodically subcultivated until transgenic shoots appear. Usually, few chloroplast genomes are transformed, and heteroplasmic cells result. Thus, these cells must be repeatedly subcultivated *in vitro* under selection pressure to eliminate non-transgenic genomes from the regenerated seedlings and to prevent the loss of the transgenic genomes (Bock, 2015). For this purpose, the use of two selectable marker genes (*aphA6* and *nptII*) improved the selection efficiency of cotton chloroplast transformation, while the *bar* gene has not been a suitable plastid-selectable marker in other crops (Verma and Daniell, 2007). Some reporter genes (e.g., *gus* and *gfp*) can be fused with recombinant proteins or independently expressed to assist with the selection of transgenic chloroplasts (Verma and Daniell, 2007). Simultaneous overexpression of chaperones has been shown to confer greater stability to recombinant proteins, reducing possible degradation by chloroplast proteases and increasing their yield.

Some limitations of this strategy still need to be overcome, such as optimization of the transformation method (e.g., transgene delivery), selection of transformed and homoplasmic cells, and plant regeneration with high efficiency for a greater number of plant species. In this context, tobacco is currently considered the best model plant to evaluate biotechnological tools by chloroplast genome transformation via organogenesis. However, in several plant species (e.g., sugarcane, rice, and *Setaria viridis*), non-green tissues (e.g., the embryogenic calli that contain only a few proplastids) are preferable for nuclear genetic transformation, while leaf tissue (which contains many chloroplasts) leads to great difficulty in regenerating transgenic plants. Therefore, for high-efficiency transformation and expression, it is critical to identify the best promoters, 5′- and 3′-UTR sequences, insertion sites (intergenic spacer regions) and selectable marker genes for specific plant species (Grevich and Daniell, 2005).

### Alternative Methods for Plant Transformation

Inducing elite transgenic events with high transformation efficiency; reduced time, cost, and labor; and reduced or absent somaclonal variation is desirable to meet the current demands of agricultural producers. For example, Manickavasagam et al. (2004) and Mayavan et al. (2013, 2015) have optimized tissue culture-free plant transformation methods mediated by *A. tumefaciens* using sugarcane axillary buds, stem cuttings or seeds. Similarly, plant transformation via pollen tubes has shown advantages, such as being genotype-independent and tissue culture-free, having high efficiency, and providing the possibility of obtaining an event without selection markers (de Oliveira et al., 2016). However, these methods are rarely used at present, possibly because they require special handling. Additional methods for *in planta* transformation, similar to pollen tube transformation, have demonstrated higher efficiency using carrier nanoparticles to efficiently deliver MCs (Grossi-de-Sa, MF; Personal communications). The root transformation and hairy root induction mediated by *A. rhizogenes* have been successfully used as a model for studies of gene expression and function in several plant species (Ron et al., 2014; Daspute et al., 2019). In addition, hairy root culture is now widely used as a bioreactor system for the production of biomolecules (Aggarwal et al., 2018). Its simplicity and high transformation efficiency have made this system an excellent method for proofs of concept.

### Clean-Gene Technology

Selectable marker genes are often indispensable for the selection of transformed cells and the production of transgenic plants. However, once transgenic plants are obtained, these elements are expendable and may be undesirable in biosafety terms. Given this, some strategies are used to minimize or avoid these problems. The simultaneous use of two or three vectors, one carrying the GOI and the others carrying the selectable marker or reporter genes, allows the generation and selection of events with both transgenes (Kumar et al., 2010), and the use of a single vector with two independent T-DNAs has enabled similar results in obtaining marker-free events (Zhu et al., 2007). In both cases, by using Mendelian segregation, it is possible to eliminate the undesirable transgene. However, these strategies, despite having promising results, have rarely been used due to their low cotransformation efficiency and because not all plant species can be effectively subjected to segregation (e.g., sugarcane and grapevine). To overcome these drawbacks, several strategies to specifically remove these undesirable elements but retain the GOIs have been developed. Some of these strategies are based on site-specific recombination systems or nucleases that mediate site-specific cleavage (Yau and Stewart, 2013). For example, the multiautotransformation (MAT) binary vector system uses oncogenes (e.g., *ipt*, *iaaM/H*, or *rol*) from *A. tumefaciens* combined with a site-specific recombination system as a selectable marker gene (Ebinuma and Komamine, 2001). For this method, the T-DNA contains two modules in tandem: (i) the GOI with a promoter and terminator and (ii) an oncogene driven by a constitutive promoter, a recombinase (R) gene driven by an inducible promoters, terminators, and two recombination sites (RSs) flanking the second module. Then, transgenic plants are regenerated, module 2 is removed by the R/RS system, and marker-free transgenic plants are selected (Ebinuma et al., 2004, 2005). Timerbaev et al. (2019) used the MAT vector system with the *nptII* selectable marker gene to successfully develop marker-free apple trees. Similarly, the FLP/FRT (Hu et al., 2006; Shan et al., 2006; Hu et al., 2008), Cre/Lox (Du et al., 2019), CINH/RS2 (Moon et al., 2011), and GIN/GIX (Onouchi et al., 1991) site-specific recombination systems have been successfully used in the generation of marker-free plants; they operate very similarly to the MAR system and show high efficiency in DNA excision. In addition to the elimination of the CRISPR/Cas9 transgene by plant segregation, the generation of transgene-free elite events has been made possible by genome editing using RNPs (Cas9 nuclease plus a guide RNA), plant regeneration in non-selective medium, and screening of plant bulks using PCR (Liang et al., 2017). Additionally, the knockout of undesirable marker genes by creating insertion/deletions (indels) or the complete removal of these genes are promising strategies using CRISPR/Cas9 (Cai et al., 2018).

## Approaches to Regulating Gene Expression

### Gene overexpression

Gene overexpression is one of the main strategies in plant functional genomics and includes both inactivating (loss-of-function) and activating (gain-of-function) mechanisms. Several GOIs associated with desirable agronomic traits have already been overexpressed in crops, and many of these GOIs have optimum activity according to their mode of expression (e.g., tissue-, stage-, or condition-specific). Therefore, GOI overexpression triggered by biotic and abiotic stress-induced promoters or tissue- or developmental stage-specific promoters has made it possible to achieve highly advantageous phenotypes with a reduced yield penalty in comparison with that of strong, constitutive expression (Kong et al., 2016; Wang et al., 2018). A case-by-case optimization of all genetic elements used is also necessary for success with this technique since overexpression alone does not guarantee the desired phenotype.

### Gene Stacking Strategy

Plants are constantly challenged by simultaneous abiotic and biotic stresses (cross-stress). In addition, the majority of agronomically important traits are under complex multigenic control and can be tuned by the effects of genes versus environment interactions (Arzani and Ashraf, 2016; Shehryar et al., 2019). Thus, the advantages of pyramiding GM traits are apparent. Two or more GOIs in the same expression cassette have been successfully used to simultaneously obtain one or more desirable agronomic traits in crop plants (Aznar et al., 2018). GOI stacking is a powerful strategy to overcome the frequent breakdown of resistance, facilitate the management of insect pests or pathogens, enhance agronomic traits, and generate elite events with multiple traits. However, different promoter and terminator sequences for each GOI are fundamental requirements for high stability of these transgenes. The size of the MC is a critical limiting factor for efficient *Agrobacterium*-mediated delivery, transgene integrity, and transformation efficiency. However, long fragments of DNA of up to ∼30 kb, containing several stacked GOIs, have been integrated into plant genomes (McCue et al., 2019). In contrast, plant artificial chromosomes are minichromosomes containing only centromeres, telomeres, and origins of replication; they are stable during mitosis and meiosis and transmitted across cells and generations (Yu et al., 2016). Minichromosomes are used as autonomous non-integrating vectors that often carry several GOIs. Carlson et al. (2007) showed the efficient meiotic transmission of an autonomous maize minichromosome. The features, advantages, and drawbacks of plant minichromosomes are discussed in detail by Yu et al. (2016).

### RNAi-Mediated Downregulation of Genes

**FIGURE 2:**
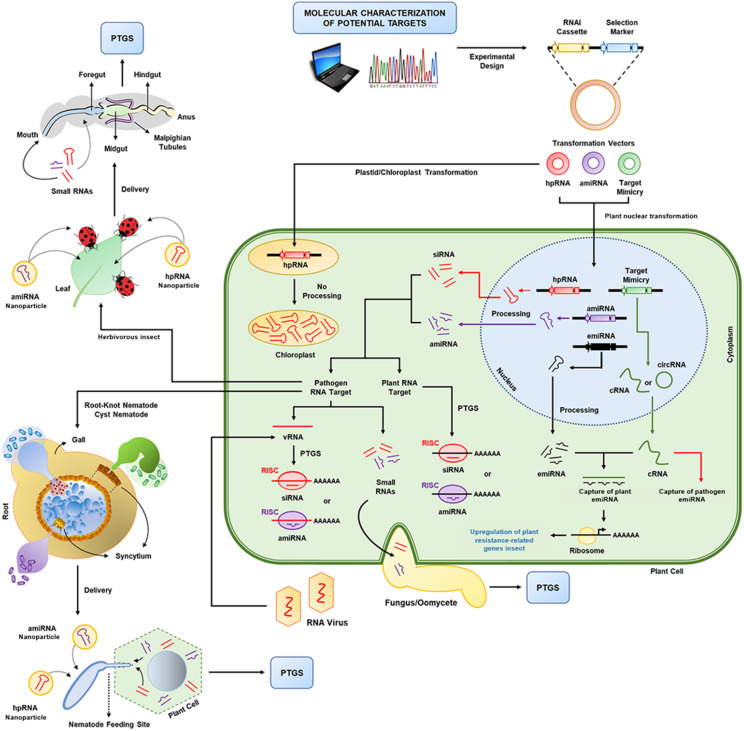
RNA interference (RNAi) technology in plants. After the molecular and phenotypic characterization of potential target genes (from plants or pathogens), expression cassettes are designed for small RNA accumulation in transgenic plants. Here, three transformation vectors for the expression of three different types of small RNAs are shown: hairpin RNA (hpRNA), artificial microRNA (amiRNA) and target mimicry molecules (miRNA sponges), which are called competitive RNAs (cRNAs) or circular RNAs (circRNAs) and can capture plant or pathogen endogenous miRNAs (emiRNAs). After nuclear transformation, these small RNAs are expressed and processed by the plant cell. The short interfering RNAs (siRNAs) and miRNAs produced combine with the plant RNA-induced silencing complex (RISC), which will promote gene knockdown in the plant (e.g., susceptibility genes) or pathogen (e.g., viruses and virulence genes). For extracellular pathogens (e.g., fungi/oomycetes), the small RNAs produced can be transferred at the site of contact between the plant cell and the pathogen. For more complex multicellular eukaryotes (e.g., insects and nematodes), the delivery of small RNAs occurs mainly through feeding. Additionally, non-transgenic approaches based on nanoencapsulation of dsRNAs or small RNAs can be applied to gene knockdown. PTGS: posttranscriptional gene silencing; vRNA: viral RNA.

The RNAi machinery in plants acts as an endogenous regulatory pathway based mainly on small interfering RNA (siRNA)-dependent gene silencing and molecular defense against any invasive nucleic acid (e.g., DNA or RNA viruses and viroids). Plants have several siRNA classes of endogenous origin (e.g., trans-acting siRNAs, natural antisense siRNAs, and heterochromatic siRNAs) encoded by repeats or intergenic regions and transposable elements, which act at the PTGS or transcriptional gene silencing (TGS) levels (Chen et al., 2018; Figure 2). The RNAi pathway is enhanced by any free double-stranded RNA (dsRNA) present in the cytoplasm; the dsRNA is immediately cleaved by ribonuclease-III-related enzymes (Dicer-like 1–4 in plants), generating several short dsRNAs (typically 20–24 nt in length). These short dsRNAs are 3′ methylated, and the guide RNA strand (antisense) combines with the RNA-induced silencing complex (RISC) and acts as siRNA, binding to the complementary mRNA. In the RISC, siRNAs bind to ARGONAUTE nucleases, and their perfect matching with the target mRNA results in either mRNA cleavage by the ARGONAUTEs or inhibition of translation. The robustness of this mechanism is dependent on the amplification of the silencing signal triggered by the siRNAs; this amplification is performed by the binding of RNA-dependent RNA polymerases (RdRPs) to the siRNA/mRNA complex, resulting in the *de novo* synthesis of dsRNA and siRNA (Borges and Martienssen, 2015).

Initially, RNAi technology was widely used in proofs of concept for functional assessment and for phenotypic characterization of genes involved in some biological processes by the loss-of-function strategy. However, it was soon found that it had enormous potential as an approach to manipulate the expression of genes associated with desirable phenotypes or agronomic traits. Currently, several RNAi-based biotechnological tools have already been developed to downregulate key genes associated with economically important agronomic traits (Rosa et al., 2018). These RNAi-based NBTs were developed by constitutive overexpression of the long dsRNA engineered in the sense/antisense orientation and separated with a spacer or intron sequence. Thus, the synthetic dsRNA presents high sequence identity with the target mRNA, leading to the successful downregulation and accumulation of target-specific siRNA. Its sense sequence should be ∼150–250 nt in length, and off-targets (sequences with high nucleotide identity with non-target mRNAs) should be avoided. The *pdk* and *adh1* introns from the *pyruvate dehydrogenase kinase* and *alcohol dehydrogenase 1* genes, respectively, have been widely used in plant RNAi technology (Ali et al., 2017). Strong and constitutive overexpression has been the most commonly used strategy to date, however, tissue- and stage-specific or stress-induced promoters can also be used according to the objective of the NBT. Typical binary vectors for RNAi technology must contain a selectable marker gene with a constitutive promoter and a classical transcription terminator sequence and the RNAi construct (target sense/antisense sequence spaced by intron sequences) with a desirable promoter sequence and a classical transcription terminator sequence. Optionally, the TRV-VIGS (*Tobacco rattle virus*) or *Agrobacterium*-mediated system can be used to transiently express the RNAi constructs in plants for reverse genetics studies. Another additional strategy is RNAi production *in planta* by a transgenic approach to control crosstalk between pathogens (e.g., nematodes and fungi) and insect pests (e.g., *Anthonomus grandis* and caterpillars). This strategy allows crops of interest to produce and accumulate siRNAs that are engineered to not regulate any endogenous gene but have as their target the organisms that attack and feed on these crops. Thus, when these organisms feed on transgenic plants, the siRNAs are ingested and taken up by the digestive system where they act on the PTGS pathway, regulating essential genes (these siRNAs can cause a curly leaf phenotype when targeted to knock out specific genes essential for the life cycle) (Ali et al., 2017). However, the dynamics of dsRNA processing and siRNA production in insects are somewhat different from those in plants (although these are still not well understood). Thus, siRNAs (20–24 nt in length) produced *in planta* are not efficient for knocking out insect genes, while full-length dsRNAs have been shown to be more efficient (Whyard, 2015; Burke et al., 2019). Burke et al. (2019) showed that long dsRNAs *in planta* produced from plastid transformation and targeting the *v-ATPaseA* gene of *Manduca sexta* led to inefficient RNAi-based insect control, suggesting that the stability and length of the dsRNA may have been affected. To overcome this problem, long dsRNA molecules were engineered *in planta* with a viroid-like structure to knock out insect pest genes. These structured dsRNAs are flanked by pH-dependent ribozyme domains, which are not processed by the RNAi machinery of plants but are efficiently processed in insect digestive tracts and cells (Macedo et al., 2017).

Recently, non-transgenic RNAi technology was optimized for the topical delivery (foliar spraying) of nanostructure-stabilized dsRNA molecules in crops for pathogen control or insect pest management (Joga et al., 2016; McLoughlin et al., 2018). dsRNA-carrier nanoparticles (e.g., biopolymers of chitosan, carbon, silicon, and clay nanosheets) (Mitter et al., 2017), RNP particles (e.g., peptide transduction domain-dsRNA binding domain) (Gillet et al., 2017), simultaneous knockout of insect digestive system nucleases (Garcia et al., 2017; Prentice et al., 2019), and cross-linkers (e.g., tripolyphosphate, dextran sulfate, and poly-D-glutamic acid) (Raja et al., 2015) were successfully developed and optimized to improve delivery and dsRNA internalization in cells, preventing dsRNA degradation and improving oral delivery to insects (Cunningham et al., 2018). Large-scale dsRNA production is still the main bottleneck of this approach (e.g., costs of production); however, there are already some private companies that supply these molecules, nanoparticles and stabilizing compounds (e.g., EZBiolab in the USA and Biomics Biotechnologies in China). Finally, all of the RNAi strategies reviewed above can efficiently knock out single or multiple target genes simultaneously. Worrall et al. (2019) showed that topical application of dsRNA assembled in layered double hydroxide nanoparticles was effective against mechanical inoculation and aphid-mediated inoculation of the *Bean common mosaic virus*.

### Fine-Tuning of miRNAs to Improve Agronomic Traits

Plant miRNAs are typically 21–24 nt in length and are transcribed in the nucleus from non-protein-coding genes (*MIR* genes). Each primary transcript (pri-miRNA) is 5′ capped and 3′ polyadenylated and forms a stem-loop structure, which is processed by Dicer-like nucleases, resulting in a pre-miRNA. These pre-miRNAs are again processed by Dicer-like nucleases, resulting in the typical duplex miRNAs, which are 3′ methylated and shuttled to the cytoplasm. Single-stranded miRNAs modulate the spatiotemporal accumulation of several target mRNAs by sequence-specific cleavage or translation inhibition (PTGS) (Borges and Martienssen, 2015). In addition, 24 nt miRNAs can return to the nucleus and mediate TGS by RNA-directed DNA methylation (RdDM) (Castel and Martienssen, 2013).

The differential expression of *MIR* genes is mostly correlated with up- or downregulation of their target mRNAs and is associated with a biological response (e.g., tolerance to drought, salinity, and nutritional deprivation) or phenotype (e.g., growth, flowering, and senescence) (Ferdous et al., 2015; Hackenberg et al., 2015). Thus, the fine-tuning of specific *MIR* genes by genetic engineering is considered a powerful biotechnological tool to improve important agronomic traits (Zhang, 2015; Teotia et al., 2016). In addition, miRNA upregulation associated with desirable agronomic traits can be achieved by overexpressing its *MIR* gene under the control of native, tissue-specific, stress-induced or developmental stage-specific promoters. However, strong and constitutive promoters often result in pleiotropic phenotypes (Basso et al., 2019). In addition, artificial *MIR* genes (amiRNAs) can be engineered in transgenic plants to produce specific miRNAs and effectively silence target genes (including endogenous or exogenous mRNAs, such as those of insect pests or pathogens). amiRNAs have sequences and structures similar to those of known *MIR* genes, except for the duplex miRNA sequence, which is replaced by a specific miRNA sequence. The selection of a backbone (pre-amiRNA sequence) for effective silencing without any off-target effects is a critical step; it must present low sequence similarity to non-target genes. Agrawal et al. (2015) showed that tobacco plants overexpressing the amiR-24 insect-specific microRNA acquired insecticidal activity against *H. armigera*.

In contrast, miRNA downregulation is also possible via genetic engineering using an artificial target mimicry (ATM) strategy. ATM is a synthetic non-coding RNA with a nucleotide sequence similar to that of the target mRNA but containing a binding site for a specific miRNA with three mismatches at the CS to prevent ATM cleavage (Banks et al., 2012). ATM acts by sequestering miRNA, and consequently, its target mRNA remains stable. Multiple miRNAs can be simultaneously downregulated using short tandem target mimics (STTMs) or SPONGES (Figure 2). STTMs contain two or more miRNA binding sites spaced by some nucleotides, while SPONGES contain multiple miRNA binding sites in tandem (Reichel et al., 2015; Thomson and Dinger, 2016). Canto-Pastor and Santos (2019) improved tomato resistance to bacterial and oomycete pathogens using STTM RNAs targeting miR482/2118.

### CRISPR/Cas9-Mediated Genome Editing

**FIGURE 3:**
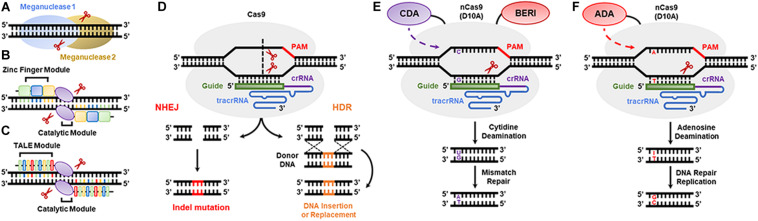
DNA genome editing techniques used in transgenic plant development. **(A)** Meganucleases, **(B)** zinc finger nucleases, **(C)** transcription activator-like effector nuclease (TALEN), **(D)** the CRISPR/Cas9 system based on non-homologous recombination system (NHEJ) and homology-directed recombination (HDR) strategies, **(E)** cytidine deaminase-based DNA base editors, and **(F)** adenosine deaminase-based DNA base editors.

Meganucleases, zinc fingers (ZNFs), and transcription activator-like effector nucleases (TALENs) (Figures 3A–C) were the first nucleases used in plant genome editing. Meganucleases recognize conserved sequences of 12–42 nt, while ZNFs consist of two modules of tandem repeat DNA-binding domains flanking the *Fok*I nuclease catalytic domain, where a binding domain recognizes a unique nucleotide triplet, and each module recognizes up to 24 nt. In contrast, TALENs also comprise two modules of tandem repeat DNA-binding motifs flanking a *Fok*I motif, but each binding domain recognizes only one nucleotide (Streubel et al., 2012). In the last 10 years, CRISPR/Cas9 or optimized nucleases (e.g., CRISPR/Cpf1 or CRISPR/Csm1) have been successfully used in plant genome editing (Osakabe et al., 2016; Wang et al., 2018). These nucleases are guided to the genome by a short RNA (approx. 20 nt in length) with a specific sequence targeting a genomic DNA sequence, and they cause double-stranded breaks (DSBs) at a target site containing a conserved protospacer adjacent motif (PAM). Consequently, the DNA repair machinery of plants can erroneously insert or delete nucleotides during DSB repair. Given this, the CRISPR/Cas9 non-homologous end joining (NHEJ) strategy was developed to introduce indels in protein-coding regions, resulting in frameshift and knockdown of the desired genes (Figure 3D). In addition, the CRISPR/Cas9 homology-directed repair (HDR) and homology and recombination-directed repair (HRDR) strategies allow nucleotide-specific editing of gene or promoter sequences using engineered synthetic donor DNA in addition to Cas9 nuclease and single-guide RNA (sgRNA) (Figure 3D; Sun et al., 2016). These three CRISPR/Cas9 strategies can be anchored in the plant genome through a transgenic approach so that the components act in *trans*, either through transient expression of the components or by direct cytosolic delivery of the CRISPR/Cas9 RNP (Lowder et al., 2015; Liang et al., 2017). The nicking variant of Cas9 (nCas9) fused to cytidine and adenosine deamination domains is also used in genome editing (Figures 3E,F). Furthermore, the mutations generated by the CRISPR/Cas9 system are stable and inheritable by classical Mendelian segregation to subsequent generations. Zhang et al. (2019) enhanced rice salinity tolerance via CRISPR/Cas9-targeted mutagenesis of the OsRR22 gene. Bao et al. (2019) showed that CRISPR/Cas9-mediated targeted mutagenesis of GmSPL9 genes in soybean improves plant architecture. Tian et al. (2018) generated an herbicide-resistant watermelon variety using CRISPR/Cas9-mediated base editing of the *als* gene.

### CRISPR/dCas9-Mediated Transcriptional Regulation

**FIGURE 4:**
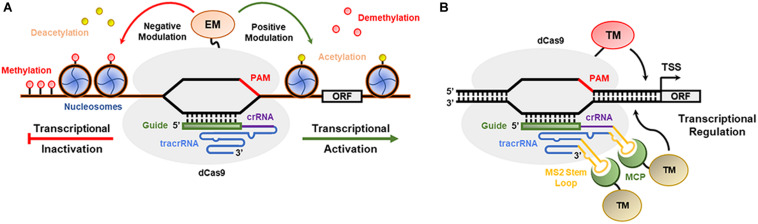
CRISPR-based epigenetic/transcriptional modulation in plants. **(A)** dCas9 combines with epigenetic modulators (EM) to modulate the formation of euchromatin and heterochromatin in plants. **(B)** A CRISPR-based transcriptional module is presented with dCas9 anchored in a gene promoter and interacting with transcriptional modulators (TMs).

The CRISPR/Cas9 and CRISPR/Cpf1 systems were engineered to modulate the transcription (activation or repression) of desired genes (Tang et al., 2017). Deactivated Cas9 nuclease (dCas9), which lacks the HNH and RuvC domains (D10A/H840A) involved in DSB production and is fused at the C-terminus to transcriptional activator or repressor domains, can be guided by a typical sgRNA to any GOI promoter sequence to modulate its expression (Figures 4A,B; Lowder et al., 2015, 2017). For example, CRISPR/dCas9:VP64 (triple or quadruple tandem repeat of the *Herpes simplex virus* VP16 activation domain) leads to transcriptional activation (Chavez et al., 2015), while dCas9:SRDX (synthetic transcriptional repressor pco-dCas9-3X) and dCas9:KRAB (Kruppel-associated box) act as strong transcriptional repressors (Lowder et al., 2017). In addition to dCas9-VP64, the fused MS2-p65-HSF1 activation domains are simultaneously overexpressed, interact with the stem loop of the sgRNA and recruit additional TFs to the promoter of the target gene (Konermann et al., 2014; Roca Paixão et al., 2019). In contrast, dCas9:SET (H3K9me3 methyltransferase domain) and dCas9:AT (acetyltransferase domain) act as epigenetic modifiers, which are expected to expand or condense chromatin to activate gene promoters (O’Geen et al., 2017). Furthermore, the dCas9-SunTag strategy is based on the fusion of dCas9 with specific tandemly repeated peptides that strongly bind and recruit other activator peptides or proteins, improving transactivation or epigenome editing (Huang et al., 2017). Tang et al. (2017) showed efficient transcriptional repression of the *miRNA159b* gene in *A. thaliana* using the CRISPR/dCpf1-SRDX system. Park et al. (2017) used the CRISPR/Cas9-V64 system and p65-HSF activators to increase the transcriptional levels of *anthocyanin pigment 1* (*PAP1*) and *vacuolar H* + *-pyrophosphatase* (*AVP1*) genes in *A. thaliana*. Papikian et al. (2019) adapted the dCas9-SunTag system in *A. thaliana* to engineer transcriptional activation with the transcriptional activator VP64 and DNA methylation with a catalytic domain from the *N. tabacum* DRM methyltransferase.

### CRISPR/Cas13a-Mediated RNA Editing

**FIGURE 5:**
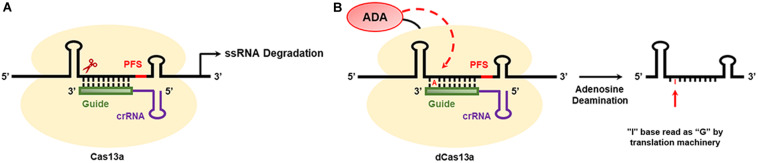
CRISPR/Cas13a-based knockdown and RNA base editing in transgenic plant development. **(A)** The CRISPR/Cas13a system can be used to degrade specific ssRNAs due to the presence of two higher eukaryotic and prokaryotic nucleotide-binding endo-RNase domains and the absence of a DNase catalytic site. **(B)** The dCas13a nuclease (with RNAse domains mutated) can be fused to specific deaminase domains to promote single-base editing. So far, the deaminase domain most successfully fused to the Cas13a nuclease was that of an adenosine deaminase (ADA), specifically, the ADAR2 domain, which is capable of converting adenosine (*A*) to inosine (*I*), which in turn is recognized as guanine (*G*) by the translation machinery, in ssRNA molecules.

Cas13a nuclease (class II type VI-A endoribonuclease) is used in the CRISPR system to target and cleave single-stranded RNA (ssRNA or mRNA) and is also dependent on protospacer flanking site (PFS) motifs (Figures 5A,B). Cas13a from *Leptotrichia wadei* (LwaCas13a) is guided by sgRNA and contains two nucleotide-binding domains (2x HEPN) associated with different RNase activities. CRISPR/Cas13a has been successfully established in mammalian and plant cells to knock down any exogenous or endogenous RNA (e.g., for immunity against viral RNA and single or multiple RNA knockdown) (Abudayyeh et al., 2017; Aman et al., 2018a). In addition, the CRISPR/Cas13a system has activity toward nuclear RNAs and has shown high target specificity. Point mutations in the HEPN domains result in disruption of its nuclease activity (East-Seletsky et al., 2017). Thus, deactivated Cas13a (dCas13a) can be fused with a deaminase domain (e.g., ADAR2 domain to adenosine-to-inosine deaminase, or dCMP domain to cytidine-to-uridine deaminase) and used to edit polymorphisms/mutations in protein-coding or non-coding RNA sequences (Cox et al., 2017). In addition, the overexpression of Cas13a or dCas13a driven by tissue-specific or inducible promoters or triggered by viral vectors can allow more precise and consistent modulation of the target RNA. Orthologous Cas13 nucleases, such as PspCas13b from *Prevotella* sp., have shown more efficient RNA knockdown than that of LwaCas13a (Cox et al., 2017). Aman et al. (2018b) used CRISPR/Cas13a to engineer *A. thaliana* for interference against the RNA genome of *Turnip mosaic virus*.

### CRISPR-Ribonucleoprotein (RNP)-Based DNA/RNA Editing

**FIGURE 6:**
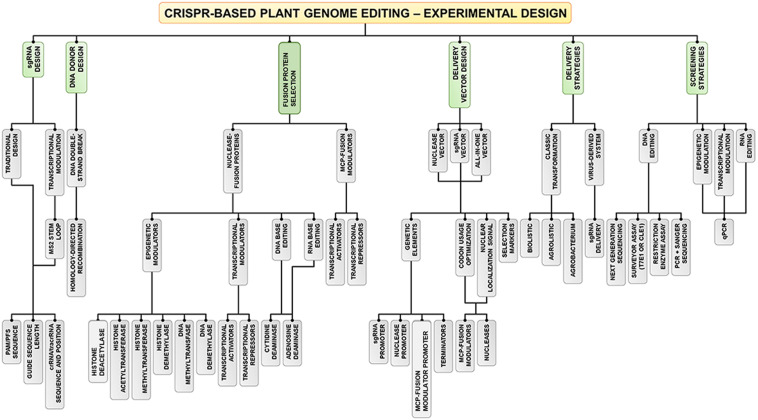
Flowchart of the suggested pipeline for CRISPR-based transgenic plant development. After a preliminary analysis of the region of the target genome that will be edited, a nuclease (Cas9, Cpf1, Cms1, Cas13a, among others) or nuclease variant (nickase or dead) should be chosen. This choice must be based on both the molecular and phenotypic responses expected from the transgenic plant, as well as on the strategy (gene overexpression, knockdown, or knockout) and the sgRNA characteristics required for each of the nucleases, such as the presence/absence and position of crRNA/tracrRNA, in addition to the PAM sequence (Cas9, Cpf1, Cms1, among others) or PFS (Cas13a).

**FIGURE 7:**
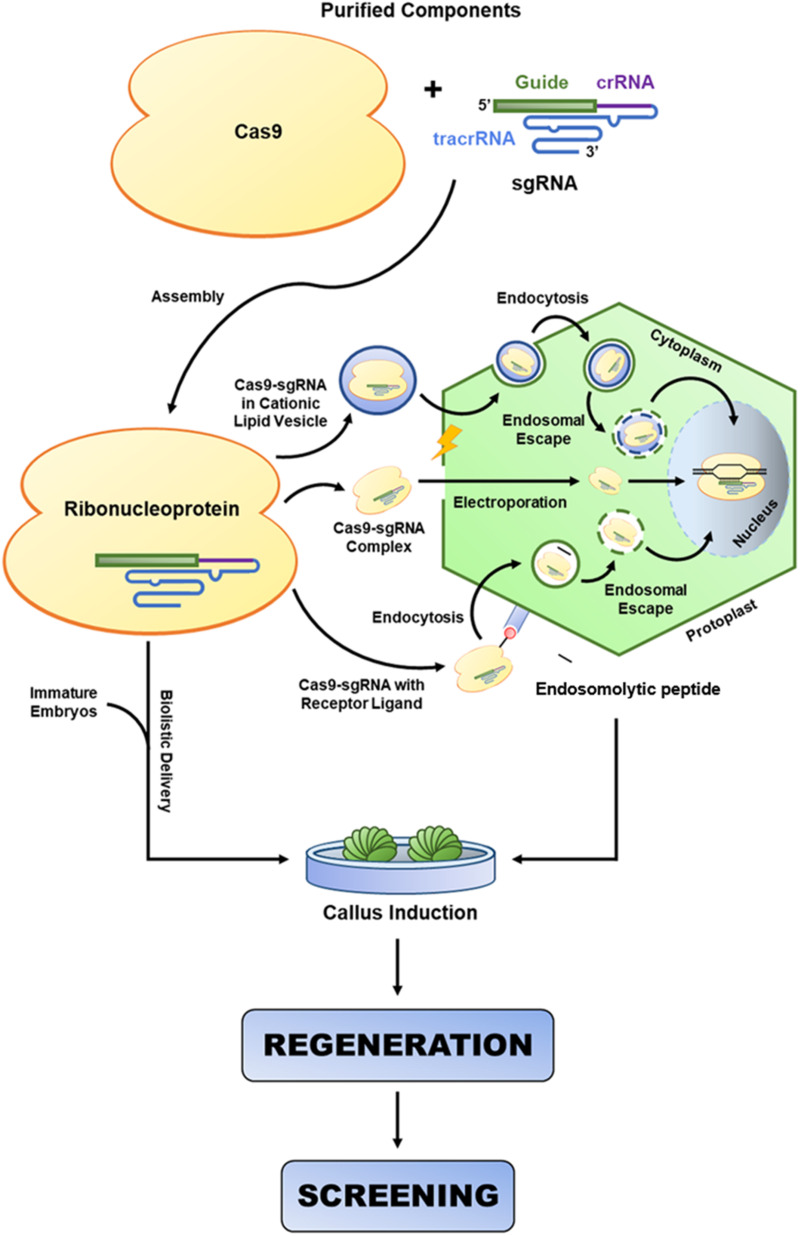
Ribonucleoprotein (RNP)-mediated plant genome editing. The experimental design starts with the design of the sgRNA, which is then transcribed *in vitro* and purified. In parallel, the Cas9 nuclease is expressed in a heterologous system and purified. After the purification and assembly of these two components (Cas9 and sgRNA), the ribonucleoprotein complex or ribonucleoprotein (RNP) can be delivered to the plant cell in several ways: (i) via electroporation; (ii) via cationic lipid vesicles; and (iii) via ligand-receptor interactions. These three RNP delivery methodologies are most commonly used in *in vitro* studies with protoplasts, and (ii) and (iii) deliver the complex via endocytosis. A fourth delivery methodology was recently presented in which the RNP is applied to coat tungsten or gold particles, and this conjugate is used to transform immature plant embryos (biolistic technique). In all these systems, after delivery, callus formation is induced for subsequent plant regeneration. Finally, the regenerated seedlings are subjected to screening steps, such as an initial PCR amplification of the DNA target region followed by Sanger sequencing and subsequent confirmation by next-generation sequencing (NGS). The advantages of this technology are that it is DNA-free and selection-marker-free, in addition to avoiding possible undesirable effects caused by constitutive Cas9 expression in the edited plants.

Advances in CRISPR-based technology have provided several strategies/pipelines for DNA/RNA editing in plants (Figure 6). The RNP technology (recombinant Cas9 associated with *in vitro* transcribed sgRNA) (Figure 7) used in plants for the acquisition of new traits can be considered the most important of these advances to date. In this procedure, there is no need to integrate any exogenous DNA into the crop genome (Liang et al., 2018). The RNPs are assembled *in vitro* and directly delivered into protoplasts or immature embryos, and cell repair mechanisms can lead to mutations in the desired target (Liang et al., 2018). Virtually all CRISPR nucleases can be produced and purified in a heterologous system (e.g., Cas9, Cpf1, Cms1, and Cas13a). The advantages of RNP-mediated editing approaches are numerous: (i) elimination of the backcrossing requirement for removal of the transformation cassette; (ii) applicability to most crops with minor adaptations (establishment and/or optimization of transfection, regeneration, and *in vitro* culture); and (iii) a small number of off-targets since the persistence of RNPs in the plant system is relatively short (∼48 h) (Kim et al., 2017). In contrast, a major drawback is editing efficiency, which is lower than that of DNA-dependent approaches (Metje-Sprink et al., 2019). Liang et al. (2017, 2018) showed the DNA-free genome editing of bread wheat with 4–5% efficiency. Svitashev et al. (2016) reported the biolistic delivery of preassembled Cas9-gRNA RNPs into maize embryo cells and the regeneration of plants with both mutated and edited alleles. Veillet et al. (2019) showed efficient transgene-free genome editing of tomato and potato using *Agrobacterium*-mediated delivery of a CRISPR/Cas9 cytidine base editor with 12.9 and 10% edited but transgene-free plants in the first generation, respectively.

## Major Existing Problems

### Transgenic Versus Non-transgenic Approaches

In 2018, more than 191.7 million hectares were planted around the world with GM crops, an increase of ∼113-fold from 1996. The United States of America, Brazil, and Argentina are the largest growers of GM crops (ISAAA, 2018); soybean (95.9 million hectares), maize (58.9 million hectares), cotton (24.9 million hectares), and canola (10.1 million hectares) are four major GM crops (Table 1). A total of 70 countries have adopted GM crops; 26 plant the crops, and another 44 import the products.

**TABLE 1:** Global area of genetically modified (GM) crops in 2018 (ISAAA, 2018).

Country	Area planted (Million hectares)	GM crops
USA	75	Maize, soybean, cotton, canola, sugar beet, alfalfa, papaya, squash, potato, apple
Brazil	50.2	Soybean, maize, cotton, sugarcane
Argentina	23.9	Soybean, maize, cotton
Canada	12.7	Canola, maize, soybean, sugar beet, alfalfa, apple
India	11.6	Cotton
Paraguay	3.8	Soybean, maize, cotton
China	2.9	Cotton, papaya
Pakistan	2.8	Cotton
South Africa	2.7	Maize, soybean, cotton
Uruguay	1.3	Soybean, maize
Bolivia	1.3	Soybean
Other countries	0.9	–
Total	191.7	

The process of developing a new cultivar with a stably inherited target trait by conventional plant breeding can take from 10 to 25 years (depending on the crop or agronomic trait), but this duration can be reduced to 7–10 years using advanced genetic engineering tools. This statement is relevant not only to the commercial aspect but also when the issue is the development of more pathogen-tolerant/resistant crops. For example, it is known that some insect pest or pathogen populations frequently break through crop resistances (Tabashnik, 2015). In addition, by conventional breeding, the segregating DNA of interest can be transferred to new sexually compatible crops, along with other undesirable DNA fragments. This problem can be minimized using genetic engineering, since a transgenic approach allows the specific introduction of one or a few specific DNA fragments between either closely or distantly related organisms. However, the high costs and long time to release of new transgenic crops are the main bottlenecks for this approach. Given these issues, the scientific community is looking for NBTs that allow the generation of elite transgene-free cultivars, such as the topical delivery of dsRNA/amiRNA using carrier nanoparticles, genome editing using DNA-free strategies, and the possible use of clean-gene technology. The United States Department of Agriculture (USDA) announced specific regulations for transgene-free crops subjected to genome editing, considering that these new crops are indistinguishable from those developed through conventional breeding methods. Similarly, Canada, Brazil, and Argentina adopted regulatory measures for new crops with edited genomes, similar to those of the USDA, but these will be evaluated case by case (e.g., with respect to the genome editing strategy used). In contrast, European countries have adopted stringent regulatory measures similar to those for conventional GM crops. Finally, although each approach has advantages and drawbacks, conventional breeding and genetic engineering can work together to potentiate the development of NBTs with new characteristics that will be safe, fast, and specific.

### Plant Tissue Culture

Tissue culture is often an important limitation in the generation of transgenic events or the efficiency of transformation since it requires a great deal of professional experience to perform (Altpeter et al., 2016). The use of specific transformation protocols or tissue culture procedures for each strategy or plant genotype is required (Wen et al., 2019). Recalcitrant genotypes require a culture medium suitable for each step of induction, cocultivation, selection or regeneration to prevent tissue oxidation and *Agrobacterium* inhibition and to enhance selection and plant regeneration. The correct handling of explants pre- and posttransformation is considered essential to improve selection, accelerate regeneration, and improve rooting. Basso et al. (2017) showed that improved culture media, subculture frequency, and type and intensity of light were fundamental for increasing sugarcane transformation efficiency. Dong et al. (2014) improved the embryogenic callus transformation efficiency of sugarcane up to 10-fold by applying an initial heat shock at 45°C, sonication and vacuum infiltration during *Agrobacterium* inoculation and callus desiccation during the cocultivation stage. Anderson and Birch (2012) addressed several critical points and presented key procedures to improve tissue culture, efficiency transformation, and sugarcane regeneration.

### Genotype-Phenotype Relationship

The molecular characterization of transgenic events is the first step in reducing the number of elite events for screening under field conditions. Events with a high transgene expression level, efficient knockdown or editing of the endogenous genes, high accumulation of foreign protein or RNA, no insertion of a backbone fragment, a low transgene copy number (thus improving transgene stability and reducing the risks of transgene silencing), inheritability and high transgene stability across generations, a desirable phenotype, and equivalent agronomic performance in greenhouse conditions are some requirements for selecting ideal events. Given this, the generation of a high initial number of independent events (100–1000 events) is required. Both the production of a large number of events in a short time and the molecular characterization of these events require considerable financial resources, infrastructure, sophisticated technology, and the availability of skilled labor. Although there are currently companies providing plant transformation services and numerous transformation events within a few months, the cost is still quite high, and these services are restricted to just a few crops. Additionally, the molecular characterization of these events can be time consuming, laborious, and costly in recalcitrant plants (e.g., cotton and woody trees). Depending on the agronomic trait of interest, phenotyping under greenhouse conditions is a limiting factor for choosing the best events (Cobb et al., 2013; Singh et al., 2019). Thus, the choice of more accurate methodologies for phenotyping is recommended. Casari et al. (2019) used thermographic analysis to confirm genotypic variation in drought response in maize. de Sousa et al. (2017) used chlorophyll fluorescence imaging of rapid light curves in maize for drought tolerance genotype discrimination. Zhao et al. (2019) thoroughly reviewed the features of crop phenotyping and addressed the advantages and drawbacks of these approaches. Overall, field screening is the most robust phenotypic evaluation and closest to the reality of the farmer. However, field screening can accommodate only a small number of events for a more detailed assessment. Additionally, authorization by the National Commission for Biosafety to conduct field trials is often rather bureaucratic and time consuming.

## Future Perspectives

Advances in functional genomics and other omics technologies over the years have revealed the biological functions and features of innumerable elements of genetic engineering. The exploration of these elements has allowed researchers to obtain a greater number of elite events in a significantly reduced time. Several GOIs are associated with agronomic traits of great economic interest, and several NBTs have been developed to overcome the main limitations present in the agricultural sector. Although the isolation, cloning, and transfer of these elements, the heterologous expression and downregulation of GOIs, and genome editing (DNA and RNA) are currently considered more accessible methods, knowledge of their advantages and drawbacks is important to better exploit their potential and for the development of powerful NBTs. The overexpression of exogenous GOIs and the up- or downregulation of endogenous GOIs driven by stage- or tissue-specific and stress-inducible promoters has allowed directed gene expression in desirable tissues, at desirable stages, or only when the plant is under certain stresses. In this way, it is possible to reduce the yield penalty in GM plants and any adverse effects in non-target organisms. However, the use of the most common tissue-specific promoters does not result in sufficient tissue expression to confer the expected phenotype, and the stress-induced promoters do not always activate transcription with sufficient speed to achieve the desired level of resistance in that condition. Therefore, the extensive characterization of GOIs, the search for new promoter sequences with higher specificity and robustness for the desired phenotype, the characterization of *cis*-regulatory elements and the validation of viral or synthetic promoters containing specific *cis*-regulatory elements in crop plants are still needed. Additionally, the use of intron or enhancer sequences to improve the transcription or translation level of the GOIs, the optimization of all elements present in the transgene DNA cassette and the application of peptide signals to target proteins to storage organelles have proven to be important techniques in plant biotechnology. For example, these findings are important for the development of NBTs to efficiently control insect pests that target a specific tissue of a host plant (e.g., cotton boll weevil, which preferentially attacks the flower buds of cotton), in which a high amount of entomotoxic protein is required (Ribeiro et al., 2017). On the other hand, the majority of agronomically important traits in crop plants are quantitatively inherited (polygenic). To date, numerous quantitative trait loci (QTLs) have been cloned to excavate major contributing candidate genes. The identification of these important genes associated with desirable agronomic traits deserves attention (Wang et al., 2019).

Plants have been successfully engineered as biofactories for synthesizing biomolecules of pharmaceutical and industrial interest; these efforts require the optimization of the abovementioned genetic elements to improve large-scale production, streamline the development of new products, and make agribusiness increasingly competitive (Mohammadinejad et al., 2019). The use of optimized entry or binary vectors and the significant advances in plant genetic transformation (both in house and by tissue culture facilities) have assisted in proofs of concept and accelerated the development of new biotechnological products. In contrast, the constitutive transformation of the chloroplast genome to overexpress heterologous proteins or accumulate RNA or dsRNA has been a powerful strategy to increase protein accumulation and improve RNA stability (Jin and Daniell, 2015). This approach has been of considerable interest with respect to elite events for the control of insect pests using RNAi and the lack of transgene transmission via pollen. However, the presence of transgenes in both crops and biofactory plants has been a limiting factor for the commercial release of these GM plants and the commercial acceptance of their feedstock and byproducts. The use of selectable marker or reporter genes is essential for the process of obtaining elite events but may not be needed when they are used commercially in field conditions. Until recently, the removal of these elements was unlikely, but now, with the new genome editing technologies, this has become quite possible (by both complete sequence removal and gene knockdown). The new genome editing tools are revolutionizing plant biotechnology and generating new alternatives to mitigate agricultural limitations. Some approaches using the CRISPR/Cas9 system or other similar nucleases (e.g., NHEJ for gene knockdown) have allowed the generation of new transgene-free plant lines, which are currently considered non-GMO, as they are essentially mutants of conventional crops. The development of NBTs that use transgene-free technologies or that have the potential to generate transgene-free elite events (genome editing) has gained prominence in recent years due to their higher potential for consumer acceptance, their lower regulatory costs than those of GM plants and their reduced impact on the ecosystem (Schiemann et al., 2019; Dalakouras et al., 2020). Overall, both basic and applied research are essential for the development of new plants that meet the needs of agriculture worldwide in reduced time with low cost and minimal undesirable effects in non-target organisms.

## Author Contributions

MB wrote the manuscript. FA provided the figures. MG, VM, MA-F, and MFG revised and provided input to the manuscript. All authors approved the final version.

## Conflict of Interest

The authors declare that the research was conducted in the absence of any commercial or financial relationships that could be construed as a potential conflict of interest.
